# Chromosomal patterns of gene expression from microarray data: methodology, validation and clinical relevance in gliomas

**DOI:** 10.1186/1471-2105-7-526

**Published:** 2006-12-01

**Authors:** Federico E Turkheimer, Federico Roncaroli, Benoit Hennuy, Christian Herens, Minh Nguyen, Didier Martin, Annick Evrard, Vincent Bours, Jacques Boniver, Manuel Deprez

**Affiliations:** 1 Department of Clinical Neuroscience, Division of Neuroscience, Imperial College London, UK; 2 University Department of Neuropathology, Division of Neuroscience, Imperial College London, London, UK; 3 Department of Human Genetics, University of Liège, Belgium; 4 Department of Neurosurgery, University Hospital, University of Liège, Belgium; 5 Laboratory of Neuropathology, Department of Pathology, University Hospital, University of Liège, Belgium

## Abstract

**Background:**

Expression microarrays represent a powerful technique for the simultaneous investigation of thousands of genes. The evidence that genes are not randomly distributed in the genome and that their coordinated expression depends on their position on chromosomes has highlighted the need for mathematical approaches to exploit this dependency for the analysis of expression data-sets.

**Results:**

We have devised a novel mathematical technique (CHROMOWAVE) based on the Haar wavelet transform and applied it to a dataset obtained with the Affymetrix^® ^HG-U133_Plus_2 array in 27 gliomas. CHROMOWAVE generated multi-chromosomal pattern featuring low expression in chromosomes 1p, 4, 9q, 13, 18, and 19q. This pattern was not only statistically robust but also clinically relevant as it was predictive of favourable outcome. This finding was replicated on a data-set independently acquired by another laboratory. FISH analysis indicated that monosomy 1p and 19q was a frequent feature of tumours displaying the CHROMOWAVE pattern but that allelic loss on chromosomes 4, 9q, 13 and 18 was much less common.

**Conclusion:**

The ability to detect expression changes of spatially related genes and to map their position on chromosomes makes CHROMOWAVE a valuable screening method for the identification and display of regional gene expression changes of clinical relevance. In this study, FISH data showed that monosomy was frequently associated with diffuse low gene expression on chromosome 1p and 19q but not on chromosomes 4, 9q, 13 and 18. Comparative genomic hybridisation, allelic polymorphism analysis and methylation studies are in progress in order to identify the various mechanisms involved in this multi-chromosomal expression pattern.

## Background

Genes are not randomly distributed and their coordinated expression is regulated by their position along chromosomes [1-3]. New mathematical approaches are therefore needed for the analysis of expression microarrays in order to identify variations in expression of spatially related genes and map them along chromosomes.  The relationship between changes in DNA copy number and variations of mRNA expression has been previously investigated (for example [4-7]) but only a few studies have used microarrays to examine large chromosomal abnormalities [7-12].

Here we propose a novel mathematical model based on single value decomposition (SVD) and Haar wavelets, named CHROMOWAVE, that detect variations in expression of spatially related gene and visualise them on chromosomes. Wavelets are a recently introduced mathematical tool for the treatment of signals with "non-stationary behaviour" [13] (e.g a hammer blow, a plane flyover noise etc.). The counterpart of the wavelet transform is the Fourier transform that achieves optimal encoding of periodic signals. The use of wavelets for data encoding, transmission and compression is now pervasive in many fields including analysis of gene sequences and functional genomics data [14]. Application to microarrays has been proposed for the analysis of light signals of microarrays plates [15-17] or to de-noise microarray time-series [18]. Only three studies applied wavelets to explore the variation in expression of gene clusters and identify their position on chromosomes [19-21]. Allen et al. [19] first adopted the wavelet transform and used smooth wavelets to study periodical patterns of mRNA expression elicited by different promoters in the *E. coli *genome. Using a supervised statistical approach, we introduced the Haar wavelet analysis for the detection of chromosomal patterns of expression in neurodegenerative diseases [20]. The same methodology was validated by Hsu et al. [22] to denoise array-based comparative genomic hybridization (array-CGH) data. Aggarwal et al [21] combined wavelets with an empirical supervised approach to analyze chromosomal expression in a set of tumour cell-lines and matched the extracted clusters with abnormal karyotypes. Their technique was limited as it allowed the analysis of only one cell-line at a time. These two studies demonstrated that wavelets have the ability to identify the areas on chromosomes where genes show similar and coherent levels of expression.

In CHROMOWAVE, the Haar wavelet model has been further refined relaxing the previous approximation of constant gene-gene distance by taking into account the variability of inter-probe distance throughout the genome. Here, we have applied CHROMOWAVE to a sample of 27 low grade and anaplastic diffuse gliomas (Table 1) and we have demonstrated its ability of extracting and visualizing large patterns of chromosomal expression that underpin meaningful biological variation and that are relevant to clinical outcome. Results were cross-validated by application of the technique to a matching data-set previously published by another laboratory [23].

**Table 1:** Clinico Pathological Data

**Case**	**Gender**	**Age**	**Tumour location**	**Karnovski score**	**Histology**	**Treatment**	**Follow up (weeks)**	**Status**
**1 – O1**	M	27	right frontal	80	Oligodendroglioma grade II	Incomplete surgical resection	884	alive and well
**2 – O20**	F	69	left temporal	80	Oligodendroglioma grade II	subtotal surgical resection, radiotherapy and chemotherapy	99	alive
**3 – O4**	F	50	right frontal	80	Oligodendroglioma grade II	complete surgical resection, radiotherapy	216	alive and well
**4 – O23**	F	59	left frontal	80	Oligodendroglioma grade II	subtotal surgical resection and radiotherapy	135	alive and well
**5 – O19**	M	24	right frontal	90	Mixed oligoastrocytoma grade II	complete surgical resection, radiotherapy	306	alive and well
**6 – O15**	M	56	left frontal	60	Mixed oligoastrocytoma grade II	complete surgical resection, radiotherapy, chemotherapy	35	dead of tumour recurrence
**7 – O10**	F	30	right frontal	70	Mixed oligoastrocytoma grade II	complete surgical resection and radiotherapy	198	alive and well
**8 – O7**	M	32	left frontal	80	Mixed oligoastrocytoma grade II	complete surgical resection, radiotherapy	643	alive and well
**9 – O8**	M	38	left temporal	90	Mixed oligoastrocytoma grade II	subtotal surgical resection, radiotherapy, chemotherapy	50	dead of tumour recurrence
**10 – O6**	F	47	left temporal	80	Mixed oligoastrocytoma grade II	complete surgical resection	143	alive and well
**11 – O16**	F	31	right parietal	80	Astrocytoma grade II	complete surgical resection	105	alive and well after 1 recurrence
**12 – AD6**	M	40	left temporo-occipital	80	Astrocytoma grade II	complete surgical resection	169	alive and well
**13 – AD9**	M	38	left frontal	90	Astrocytoma grade II	complete surgical resection	136	recurrence
**14 – AD10**	M	70	left temporal	80	Astrocytoma grade II	complete surgical resection and radiotherapy	90	recurrence (PET Scan)
**15 – AD11**	M	25	left temporal	80	Astrocytoma grade II	subtotal surgical resection	137	recurrence (PET scan)
**16 – AD12**	M	34	left temporal	80	Astrocytoma grade II	complete surgical resection	25	alive and well
**17 – O2**	F	26	right frontal	80	Anaplastic Oligodendroglioma	complete surgical resection, chemotherapy	310	dead of tumour recurrence
**18 – O3**	M	21	right frontal	90	Anaplastic Oligodendroglioma	subtotal surgical resection and chemotherapy	867	recurrence
**19 – O17**	F	24	left temporal	80	Anaplastic Oligodendroglioma	complete surgical resection and chemotherapy	578	alive and well
**20 – O18**	F	45	right parietal	80	Anaplastic Oligodendroglioma	subtotal surgical resection and chemotherapy	105	dead of tumour recurrence
**21 – O9**	F	46	left parietal	80	Anaplastic oligodendroglioma	complete surgical resection and chemotherapy	624	dead of tumour recurrence
**22 – O24**	M	57	left fronto-temporal	80	Anaplastic Oligoastrocytoma	complete surgical resection and radiotherapy	15	alive and well
**23 – O12**	F	27	left temporal	60	Anaplastic Astrocytoma	complete surgical resection and radiotherapy	6	dead of cerebral venous thrombosis
**24 – AA3**	F	25	right frontal	80	Anaplastic Astrocytoma	complete surgical resection, chemotherapy	307	dead of tumour recurrence
**25 – AA6**	F	36	left fronto-temporal	80	Anaplastic Astrocytoma	subtotal surgical resection and radiotherapy	447	alive and well
**26 – AA5**	M	44	right temporal	90	Anaplastic Astrocytoma	subtotal surgical resection and chemotherapy	74	alive and well

## Results

**Figure 1:**
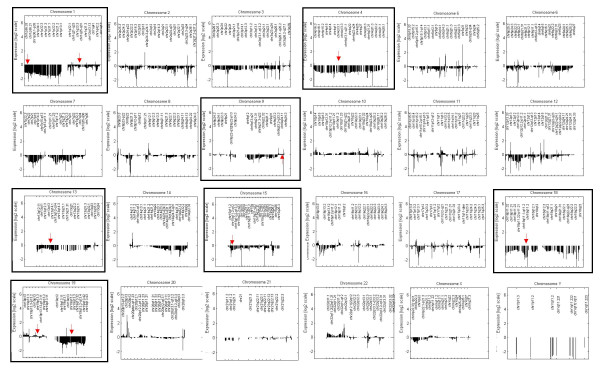
**Main pattern of chromosomal expression extracted by CHROMOWAVE**. This pattern corresponds to the main eigenvalue extracted by CHROMOWAVE for the tumour data-set. X axis represents the genomic distance along each of the 24 chromosomes and the Y axis represents the gene expression contribution (intensity and direction, log2 scale). Chromosomes on which similar levels of gene expression are seen along the entire chromosome (chromosomes 4, 18, 13, 15) or a chromosome arm (chromosomes 1p, 19q) are shown in a dark frame. Red arrows point to the location of the FISH probes used for structural analysis.

**Figure 2:**
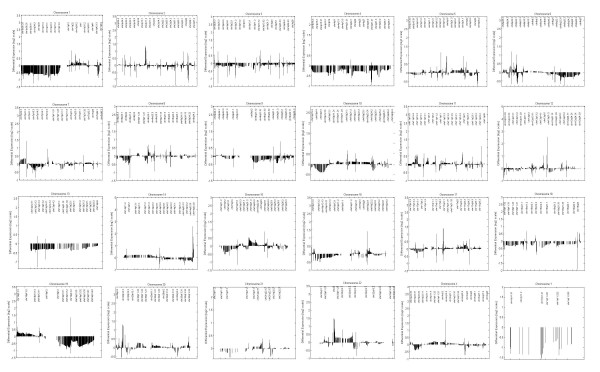
**Single Case Analysis**. This is a representative example of chromosomal expression for a single case (O10) extracted by CHROMOWAVE by contrasting its chromosomal mRNA distribution with that of a normal data-base. X axis represents the genomic distance along each of the 24 chromosomes and the Y axis represents the gene expression contribution (intensity and direction, log2 scale).

When applied to the data-set containing the 27 tumour cases, the first pattern generated by CHROMOWAVE (40% of the overall variance) consisted in a multi-chromosomal pattern of variation that revealed considerably reduced gene expression in large regions of chromosomes 1p, 9q, and 19q and of the whole chromosomes 4, 13, 15 and 18. Smaller clusters of differentially expressed genes were also observed on the other chromosomes, particularly 2, 3, 5, 7, 12 and negligible variations were present on chromosomes 8, 20, 21, and Xp. This pattern is illustrated in Figure 1 and relative data are contained in the Additional file 1. Remaining patterns accounted for < 10% of the data variability and were not considered. Note the clean display of the profiles that are completely de-noised. Figure 2 illustrates the individual profile for case O10 extracted using the supervised technique previously developed [20](see below for discussion of this particular profile).

For this data-set, FISH analysis demonstrated various combinations of monosomy on chromosomes 1p, 9q, 4, 13, 15, 18 and 19q, (Table 2).

**Table 2:** Chromowave and FISH results for the glioma data-set.

**Case**	**Diagnosis**	**FISH Load Chr1p36**	**Chromowave Load Chr1 (+ = loss)**	**FISH Load Chr4c**	**Chromowave Load Chr4 (+ = loss)**	**FISH Load Chr9q34**	**Chromowave Load Chr9 (+ = loss)**	**FISH Load Chr13q14**	**Chromowave Load Chr13 (+ = loss)**	**FISH Load Chr15c**	**Chromowave Load Chr15 (+ = loss)**	**FISH Load Chr18c**	**Chromowave Load Chr18 (+ = loss)**	**FISH Load Chr19q13**	**Chromowave Load Chr19 (+ = loss)**	**Gender**	**Chromowave Load ChrY (+ = loss)**
														
**1**	**OII (O1)**	**Mono**	**0.21**		**0.03**		**0.06**		**0.08**		**0.06**		**0.02**	Mono	**0.37**	M	-0.20
**2**	**OII (O20)**	**Mono**	**0.17**		-0.15		**0.25**		-0.22		-0.18		**0.40**	Mono	**0.20**	F	0.22
**3**	**OII (O4)**	**Mono**	**0.09**		0.00		-0.01		-0.01		-0.16		**0.03**		**0.03**	F	0.14
**4**	**OII (O23)**		**0.04**	Mono	0.00		-0.19		-0.18		-0.22		-0.17	Mono	**0.02**	F	0.17
**5**	**OAII (O19)**		**0.09**		-0.22		-0.18		-0.32		-0.14		-0.19	Mono	**0.16**	M	-0.28
**6**	**OAII (O15)**		-0.17		-0.15		**0.42**		-0.19		-0.12		-0.08		-0.51	M	-0.22
**7**	**OII (O10)**		**0.25**		**0.15**		**0.14**	Mono	**0.26**		**0.03**		**0.14**		**0.2**	F	0.18
**8**	**OAII (O7)**		-0.04		**0.06**		**0.07**	Mono	**0.30**	Mono	**0.11**		-0.02		**0.01**	M	-0.24
**9**	**OAII (O8)**		-0.19		-0.12	Mono	**0.08**	Mono	**0.03**		-0.24		-0.19		-0.22	M	-0.16
**10**	**OAII (O6)**		-0.06	NA	**0.20**		-0.01		**0.04**		**0.1**		**0.05**		**0.1**	F	0.16
**11**	**OII (O16)**		-0.08		-0.12		**0.12**		-0.08		-0.01		**0.06**		**0.05**	F	0.23
**13**	**AII (AD6)**		-0.08		-0.01		-0.12		-0.12		**0.03**		-0.02		-0.12	M	-0.20
**14**	**AII (AD9)**		-0.12		-0.04		-0.07		-0.04		**0.01**		-0.07		**0.07**	M	-0.22
**15**	**AII (AD10)**		-0.15		-0.12		-0.23		-0.22		-0.1		-0.17		-0.13	M	-0.20
**16**	**AII (AD11)**	**Mono**	**0. 1**		**0.12**		**0.12**		**0.12**	Mono	**0.27**		**0.09**		**0.01**	M	-0.19
**17**	**AII (AD12)**		-0.11		-0.09		-0.01		-0.14		-0.01		-0.13		-0.12	M	-0.21
**18**	**OIII (O2)**	**Mono**	**0.09**		**0.28**	Mono	**-0.23**	Mono	**0.27**		-0.22		0.00	Mono	**0.03**	F	0.20
**19**	**OIII (O3)**	**Mono**	**0.33**	Mono	**0.64**		**0.14**	Mono	**0.48**	Mono	**0.05**	Mono	**0.68**	Mono	**0.06**	M	0.01
**20**	**OIII (O17)**	**Mono**	**0.27**		-0.05		**0.17**	Mono	***-0.10***	Mono	-0.08		-0.07	Mono	**0.4**	F	0.18
**21**	**OIII (O18)**	**Mono**	***-0.16***		-0.15		-0.13		-0.17	Mono	**0.22**		-0.16		-0.36	F	0.13
**22**	**OIII (O9)**	**Mono**	**0.31**		**0.19**		**0.23**		**0.11**	Mono	**0.7**		**0.09**	Mono	**0.02**	F	0.19
**23**	**OAIII (O24)**		-0.15		-0.14		-0.17		-0.14	Mono	**0.03**		-0.17		-0.19	M	-0.22
**24**	**OIII (O12)**		-0.17		**0.29**		-0.04		**0.30**		0.25		**0.19**		**0.03**	F	0.13
**25**	**AIII (AA3)**		-0.15		-0.10		-0.48		**0.02**		-0.11		-0.26		-0.1	F	0.25
**26**	**AIII (AA6)**		-0.07		-0.04		**0.32**		**0.15**		-0.11		**0.09**		-0.03	F	0.15
**27**	**AIII (AA5)**		-0.05		-0.30		-0.10		-0.10		-0.08		-0.04		**0.09**	M	-0.18

This table illustrates the combined variation of gene expression of chromosomes 1p, 4, 9p, 13, 15, 18, 19q detected by CHROMOWAVE and the variations detected by FISH analysis. Note that the case-loadings reflect the normalization to the average that is performed by the SVD. Therefore, the more positive the case loading is the greater is the loss. As a rule of thumb loadings close to or grater than zero indicate monosomy. Positive cases are highlighted in bold. Cases underlined are hyperploid and cases highlighted in italics showed a discrepancy between FISH and CHROMOWAVE (loss detected in FISH not detected by CHROMOWAVE) – O: Oligodendroglioma – OA: Oligoastrocytoma – A: Astrocytoma – AA: Anaplastic astrocytoma; II grade II, III grade III – NA: not available.

We then compared FISH measurements to the individual chromosomal expressions extracted by CHROMOWAVE. When applied to one chromosome at a time, the SVD extracted as main components (> 70% of the total variability) chromosome wide diffuse signals on chromosomes 4, 9, 13, 15 and 18 and diffuse homogeneous expression on the chromosomal arms 1p and 19q. Case loadings for these patterns are displayed in Table 2. Note that the case-loadings in Table 2 reflect the normalization to the average that is performed by the SVD. Therefore, the more positive the case loading is the more the pattern is expressed (in this case the bigger the loss), the more negative the loading the less the pattern is expressed. When we compared the FISH measurements with these case-loadings we observed significant association for chromosome 1p (Pearson correlation R = 0.522, *p *= 0.005) and 19q (Pearson correlation R = 0.392, *p *= 0.043) indicating that loss of genetic material was the main cause of the reduction of expression seen by CHROMOWAVE.

Interestingly, one oligodendroglioma (case 010, see figure 2) clearly demonstrated low expression 1p/19q with CHROMOWAVE but no structural loss could be detected by FISH (Table 2). The reduced mRNA expression detected by CHROMOWAVE on chromosomes 4, 9q, 13, 15 and 18 was less commonly associated with chromosomal alterations seen by FISH (Table 2). In particular, six tumours showed low expression of 9q without alteration detected by FISH and among the seven lesions demonstrating low expression in chromosome 18, only one showed monosomy.

The discrepancy between FISH counts and expression data raises several hypotheses. First, this may be inherent to the FISH method where the probe targets only a short DNA sequence on the chromosome and is not informative of possible large losses of genetic material in regions flanking the probe target. The chromosomal areas targeted by the FISH probes are shown on Figure 1. Second, alternative genetic and epigenetic mechanisms can cause expression changes in adjacent genes in the absence of chromosomal loss such as translocation, uniparental disomy or methylation/acetylation silencing, all frequently reported in cancer. Third, hyperploidy which is frequently seen in malignant gliomas may also account for some of these observations.

**Figure 3:**
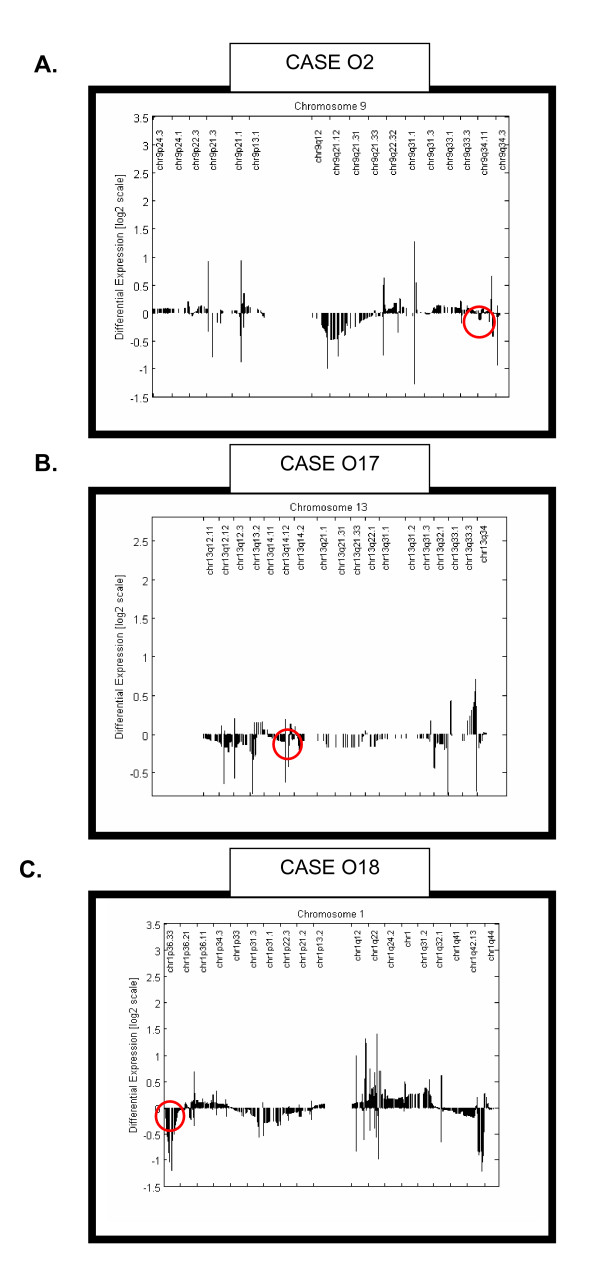
**Mismatches Between CHROMOWAVE and FISH**. This figure shows the three cases illustrated in Table 2 where FISH LOH was not matched by chromosomal expression loss estimated by CHROMOWAVE. These mismatches can be explained by the localized losses of expressions detected by CHROMOWAVE that were around the loci targeted by the FISH probes (red circles). This indicates that in these cases FISH measurements reflected localized and not chromosome wide structural deficits or anomalies.

Conversely, in three cases, CHROMOWAVE case loading was quite negative while FISH demonstrated loss of genetic material (Table 2). However, inspection of individual cases (Figure 3) allowed the following observations. In case 02, CHROMOWAVE did not display a chromosome wide reduction but in 9q.34.1 showed a region of 825 Kb with selective loss of expression around the ASS gene that is targeted by the FISH probe (Figure 3A). For case O17, CHROMOWAVE showed loss of expression but restricted to small clusters including a telomeric region of 1.526 Mb in chromosome 13q14 before and around the RB gene where the FISH target is located (Figure 3B). Case O18 also had loss of expression on 1p but restricted to a telomeric segment of 10 Mb that included the chr1p36.32 locus targeted by the FISH probe (Figure 3C). This finding suggests that in these cases FISH recognized small alterations and not large structural anomalies that were instead identified by CHROMOWAVE.

When we tested the case loadings identified by CHROMOWAVE with outcome (tumour recurrence and patient survival) using Cox regression, we found that the pattern in Figure 1 was significantly predictive of favourable outcome (*p *= 0.007). By testing each of the chromosomes and their various combinations we then observed that the covariation of chromosomes 1p, 13 and 18had the strongest correlation with survival (*p *= 0.002).

In contrast, the major gene expression pattern obtained with the same SVD analysis but without Haar wavelet transformation did not correlate with survival (p = 0.802) suggesting that the distribution of gene expression changes on chromosomes is more relevant to tumour behaviour than their raw variations of amplitude.

### Sensitivity to Individual Cases

In order to verify the stability of the pattern and its dependency upon single cases, we performed a jack-knife test to verify that association remained significant with the exclusion of single cases. The genome-wide SVD analysis was repeated after removal of one case (27 iterations), the chromosomal pattern was extracted and stored, and the correlation of the resulting case loadings with survival re-calculated using Cox regression, This process generated 27 *p*-values. The 95% confidence interval of the empirical *p*-value distribution obtained was calculated by normal approximation of the log-transformed *p*-values. Throughout the 27 permutations, the global patterns recovered were indistinguishable from that obtained with all cases, all the 27 patterns were significantly associated with outcome (*p *< 0.05) and resulting *p*-values were tightly distributed (*p*-values 95% confidence interval was [0.0372–0.0022]).

### Sensitivity to Chromosome Biology: Chromosome Y and Gender Sensitivity

**Figure 4:**
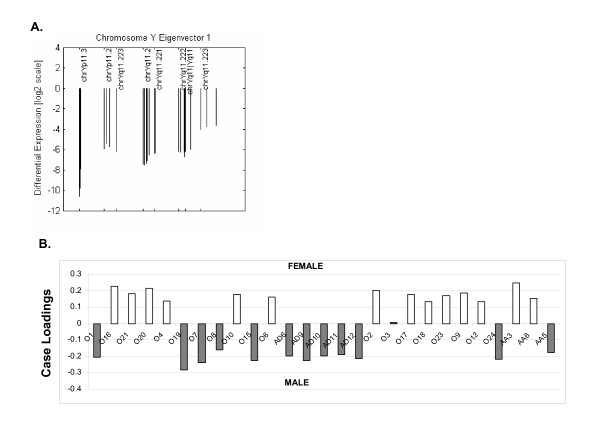
**Chr Y Analysis and Sensitivity to Gender**. **A) **The SVD decomposition of Chr.Y alone determined a uniform pattern spanning the entire chromosome. **B) **Illustration of the corresponding case loadings indicating the amount of expression of the chromosomal pattern expressed by each case. In this convention, positive values indicate loss of expression. Dark bars indicate male cases while white bars indicate female cases. There is an obvious and clear separation between arrays due to gender although a loss of Y expression is detected in tumour tissue for case O3.

For validation purposes, CHROMOWAVE was applied only to the probes of chromosome Y only for the 27 tumour cases. The main pattern of variation was extracted and the association between resulting case loadings and gender was tested by means of a Student t-test. The main component extracted by the algorithm, which accounted for 94% of the variability, was a uniform pattern on the chromosome (Figure 4A). The corresponding case loadings are shown in Figure 4B and illustrate the perfect separation of arrays according to gender (p < 10^-5^). Numerical values for the loadings are also contained in Table 2.

### Sensitivity to Denoising Parameters

**Figure 5:**
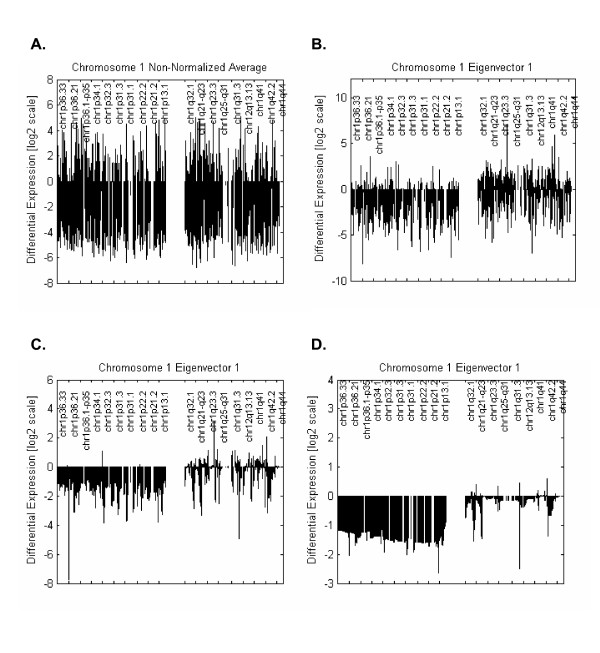
**Denoising Step-by-Step**. Demonstration of the contribution of each step of the analysis for pattern extraction on Chr.1 for the glioma data-set. **A) **Average profile of expression for Chr 1 for all the 27 tumour cases. **B) **Main pattern of expression (81% of total variability) detected by SVD. **C) **Effect of de-noising on the SVD pattern. **D) **Additional noise removal by inter-probe distance penalization.

The settings used in this work (choice of a redundant wavelet transform, statistical threshold at Eq.(5), inter-probe distance penalization at Eq.(6)) were chosen at the very conservative end of standard wavelet methodology with the deliberate aim to minimize false positives at the expense of sensitivity. Figure 5 illustrates the incremental effect of the applied methodology (SVD, denoising, inter-probe distance penalization) to the analysis of Chr.1 for this data-set. Note the ability of the technique to render a clean profile for the Chr1p anomaly that is common in the types of gliomas considered here. Importantly, the additional penalization for inter-probe distance (Fig. 5d) removes entirely the remaining spikes on Chr1p rendering a clean and biologically sensible profile.

**Figure 6:**
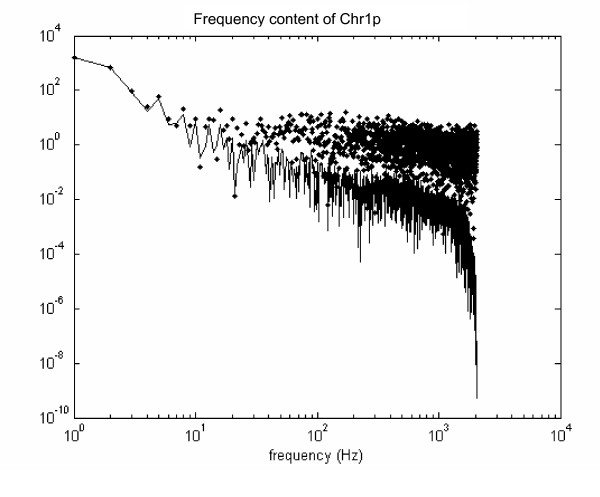
**Comparison with Fourier Power Spectrum**. Power spectrum of the raw pattern extracted by SVD for Chr1p (see Fig. 5B) (dotted) and that of the de-noised spectrum (see Fig. 5D) (line). Note that the de-noising procedure effectively removes the noise at high frequencies but preserves the large structure in the signal (< 10 Hz). The dip in the de-noised spectrum at > 10^3 ^Hz is due to the zeroing of the highest frequency content in the WT corresponding to the single probes expression.

As independent validation of the pattern extracted by CHROMOWAVE on Chr.1, we calculated the power spectrum of the ordered probes on Chr1p. The spectrum of the raw data and the one of the de-noised pattern are illustrated in Figure 6. Note that the de-noising procedure removes the noise in the high frequencies but preserves the large structure in the signal that is obviously present at frequencies of less than 10 Hz. The power spectrum calculation adopts the FFT and is based on the assumption of equidistant probes. This assumption is relaxed in CHROMOWAVE.

### Sensitivity/Specificity Analysis

Although the technique presented here is unsupervised, its sensitivity/specificity can be evaluated through simulation studies by assuming the signal distribution known. The main problem with simulations in this context is the faithful generation of the noise covariance structure of chromosomal expression that is unknown. To recover the noise covariance we have selected Chr1 that has evident Chr1p loss pattern in this data-set. We have removed from the data-set this specific monosomy by zeroing the first singular component. The remaining singular components had Morgera's Complexity ~1 [24] indicating that all that was left was noise. We have therefore built a simulation by adding a telomeric pattern of various intensities (when signal was 0 the specificity was obtained) and of varying spatial dimensions (500 Kb, 1.5 MB, the whole "petit" arm and the whole chromosome) to 13 of the 27 arrays. In the second simulation we maintained a Chr1p loss pattern but varied the number of arrays to which the pattern was added.

**Figure 7:**
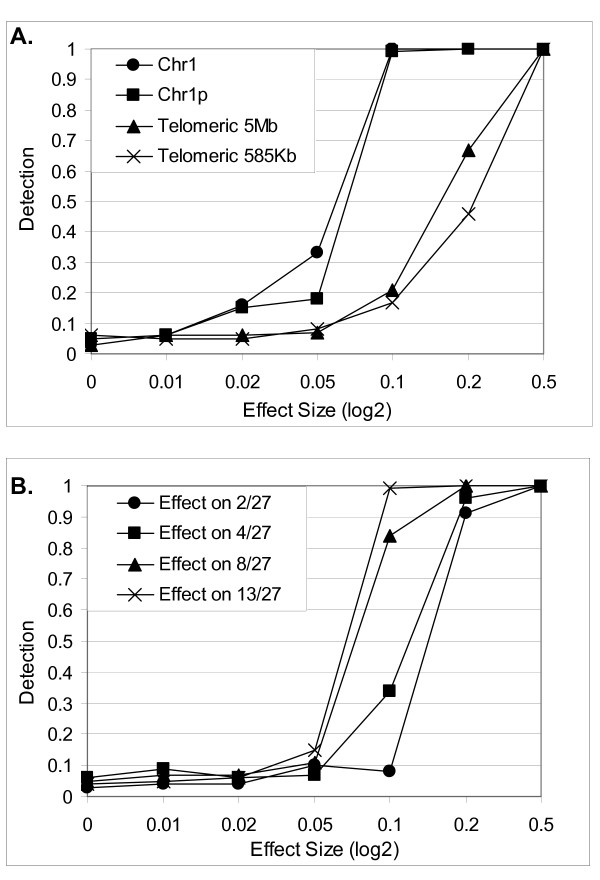
**Sensitivity/Specificity Analysis**. Results of the simulations obtained by adding an artificial pattern on Chr1 real noise. **A) **Detection is shown for varying intensities of the pattern for 4 different patterns (2 telomeric 1p patterns of differing length, than a whole 1p and a whole chromosome pattern). Specificity is measured as detection at intensity 0. **B) **The four detection lines correspond to 4 simulation studies where the pattern was added to 2, 4, 8 and 13 out of the 27 arrays in the data-set.

The detection/specificity measure was obtained by generating, for each intensity, 100 permutations of the arrays and therefore adding the signal to a random sub-set. At each iteration, CHROMOWAVE extracted the first eigenvector of the wavelet transformed data and a Student's T-test (2 tails, α = 0.05) was performed between the two groups of arrays (with and without signal). The detection metric was calculated as the number the null-hypothesis rejected divided by the number of permutations. Results for the 2 simulations are shown in Figure 7 in terms of detection versus intensity. The latter is shown in log2 scale (0.5 corresponds to a 40% increase in expression, 0.05 to a 4% increase etc.). In general terms, any pattern change greater than ~0.2 (15%) is detected with probability 1 whatever the size or the sub-set of arrays it affects. Specificity was always below or equal the specified limit (0.05) in all conditions tested confirming that noise distribution adhered to the assumptions.

### Clinical Reproducibility: Application to Freije et al. (2004) dataset

Final validation was performed by assessing the reproducibility of the association between chromosomes 1p, 13 and 18 and outcome in a published set of arrays of comparable tumour types. The only comparable data-set that was publicly available at the time of writing was published by Freije et al. [23]. Microarray files (Affymetrix HG U133A and B oligonucleotide arrays) and clinical data were downloaded from the authors' website [25]. We excluded glioblastomas from the set and we examined the 25 arrays that included 10 anaplastic oligodendrogliomas, 8 anaplastic astrocytomas and 7 mixed anaplastic oligo-astrocytomas. Microarray files were pre-processed and normalized using the same procedure adopted for our data and then entered into CHROMOWAVE. Chromosomal expressions were obtained by application of the SVD to each chromosome independently. As in our data-set, diffuse patterns of expression were found in chromosomes 1p, 4, 9, 13, 18 and 19q. Case loadings for chromosomes 1p, 13 and 18 were then entered into a Cox regression model to test their association with survival that resulted being remarkably strong (p = 0.0028) and similar to what found in our data-set.

## Discussion

CHROMOWAVE allows the unsupervised identification of clusters of adjacent genes with homogeneous changes of expression and their mapping on chromosomes, resulting in the display of multi-chromosomal gene expression patterns. Here we have demonstrated that these patterns are reliable, reproducible and statistically robust but that they are also clinically relevant. In this application, the SVD was the method of choice for statistical analysis in wavelet space: however this approach is amenable to treatment with any other unsupervised technique (independent component analysis, clustering etc.).

Low grade and anaplastic diffuse gliomas represent an interesting model to explore the variation of expression of spatially related genes rather than their individual expression. Characteristic genetic and epigenetic alterations have been found that predict favourable outcome in some tumour histological subtypes while being non informative or even carrying a poor prognosis in others. For instance, the extensively investigated allelic loss of chromosomes 1p/19q or the hypermethylation of the promoter of the drug-resistance gene O6-methylguanine-DNA methyltranferase are associated with chemosensitivity and therefore longer survival in a subset of oligodendrogliomas [26-30] but have unclear prognostic value or even correlate with more aggressive behaviour in astrocytomas and mixed oligoastrocytomas [28]. Moreover, no strong candidate tumour suppressor/promoter gene has been singled out yet on these frequently lost chromosomal segments [31]. A few studies have used expression microarrays to investigate diffuse gliomas [9,23,32-41] but none explored the expression change of gene clusters that could be relevant to tumour progression with regards to their distribution on chromosomes.

In our microarray dataset, derived for a group of 27 WHO grade II and III gliomas, CHROMOWAVE generated a multi-chromosomal pattern of variation that correlated with outcome. The pattern included diffuse losses on 1p and 19q but also on 4, 9q, 13 and 18. Among these, changes on 1p, 13 and 18 had the strongest correlation with survival. This finding was replicated on a comparable set of microarray data previously published by another laboratory [23].

Notably, while the main pattern of chromosomal variation extracted by the SVD correlated with survival, the main pattern of RNA variation extracted by the SVD from the raw data (no spatial transform applied) did not. This suggests a major role of chromosomal RNA modulation in tumour behaviour as opposed to the variation of the bulk of gene expression.

FISH studies suggested that, in our dataset, low expression on chromosomes 1p and 19q were most often the consequence of large allelic loss in these regions, an alteration commonly seen in oligodendrogliomas. However, FISH counts could not explain the diffusely reduced expression on 4, 9q, 13 and 18 raising several hypotheses: genetic loss occurring in regions flanking the FISH probe targets, genetic changes that do not result in gene loss detectable by FISH such as translocation and uniparental disomy, or epigenetic alterations such as methylation-based gene silencing. Clearly, extensive ancillary studies are needed to determine the various mechanisms underlying the CHROMOWAVE gene expression patterns.

Comparative genomic hybridisation (CGH), allelic polymorphism analysis and methylation studies are currently in progress in our laboratory. Whatever the causative mechanisms, the finding that large gene expression changes in chromosomes 4, 9q, 13 and 18 occur frequently in grade II and III diffuse gliomas and that they bear prognostic information is novel.

## Conclusion

In conclusion, we propose a new mathematical model that has proved powerful in our dataset for detecting and mapping to chromosomes biologically meaningful gene expression changes. The possibility of visualising changes of spatially related genes and their position on chromosomes make CHROMOWAVE a valuable screening method to explore microarray datasets. The mechanisms contributing to these expression patterns are probably multiple and complex. Additional studies combining FISH, CGH/aCGH, allelic polymorphism and methylation analysis are clearly needed and should target those chromosomal areas identified by CHROMOWAVE as supporting clinically relevant gene expression changes.

## Methods

We studied a dataset generated with Affymetrix U133_Plus_2 arrays (Affymetrix, Santa Clara, CA) in 27 low grade and anaplastic diffuse gliomas (clinicopathologic features are summarised in Table 1) and 11 samples of normal brain obtained in course of surgery for intractable epilepsy. Tissues were collected under the approved guidelines of the Ethics Committee of the Faculty of Medicine, University of Liège, Belgium and all patients gave informed consent for their participation in this study.

### RNA extraction, target preparation and microarray hybridisation

Total RNA was extracted from cryostatic sections using the Qiagen RNeasy kit (Qiagen, Chatsworth, CA). The integrity of the RNA was confirmed with the Agilent Bioanalyser using the RNA 6000 Nano kit (Agilent). We used the GeneChip^® ^Expression 3' Amplification One-Cycle Target Labeling kit (Affymetrix, Santa Clara, CA) to label the RNA following the manufacturer protocol. The cRNA was hybridized to Affymetrix Human U133_Plus_2 arrays according to the manufacturer protocol. Briefly, double-stranded cDNA was synthesized routinely from five micrograms of total RNA primed with a poly-(dT) -T7 oligonucleotide. The cDNA was used in an *in vitro *transcription reaction (IVT) in the presence of T7 RNA polymerase and biotin-labelled modified nucleotides during 16 hours at 37°C. Biotinylated cRNA was purified and then fragmented (35–200 nucleotides), together with hybridization controls and hybridized to the microarrays for 16 h at 45°C. Using the Fluidics Station (Affymetrix), the biotin-labeled cRNA was revealed by successive reactions with streptavidin R-phycoerythrin conjugate, biotinylated antistreptavidine antibody and streptavidin R-phycoerythrin conjugate. The arrays were finally scanned in an Affymetrix/Hewlett-Packard GeneChip Scanner 3000

### Preliminary data analysis

Preliminary data analysis was conducted using the software of the Affymetrix microarray suite (MAS, version 5.0) following the statistical procedures described in the Affymetrix: Statistical Algorithms Detection Document [42]). MAS produced an expression value plus an index parameter indicating positive or negative detection (present call index) for each of the 54,675 probe sets on the chip (settings used were standard for the U133_Plus_2 array: alpha1 = 0.05, alpha2 = 0.065, Tau = 0.015, TGT = 100). Statistical analysis and post-processing were performed using an in-house software (CHROMOWAVE) written in MATLAB 6.5 (The Mathworks Inc., Natick MA, USA). Individual arrays were normalized to the background by dividing intensities by the median value of those genes presented with positive detection. Expression values where then log2 transformed.

### Mapping Target Sequence Values to Chromosomal Location

Expression values were mapped to their corresponding chromosomal location and then sorted within each vector using genome alignment information. Information on the physical location of each gene and the respective genome alignment information for each target sequence on the HG-U133_Plus_2 chip were obtained from the Affymetrix website [43].

### Haar Wavelet Analysis of Chromosomal Expression

Gene expression values were analysed through CHROMOWAVE that uses the positional information of genes and statistical analysis to extract chromosomal pattern of gene expression. CHROMOWAVE applies the wavelet transform (WT) to the spatial distribution of the array probes and converts the original expression values in wavelet coefficients that are functions of the expression of adjacent genes. Wavelet coefficients are then filtered so that only those with high signal-to-noise ratio and/or representing probes with close genomic distance are retained. The application of the inverse WT produces a noise-free pattern of chromosomal gene expression. When multiple arrays are used, wavelet coefficients can be used in statistical analysis in the same fashion as gene expression values either with supervised methods of analysis (t-tests, ANOVA, discriminant analysis, etc.) or unsupervised (clustering techniques, independent component analysis etc.). The WT algorithm has been described elsewhere (see for example [44]) and will be only summarized here. The traditional WT scheme is limited by the decimation step that may "miss" relevant signal elements, particularly when noise levels are high as in this application. For this reason, CHROMOWAVE adopts the "cycle spinning" WT that has greater complexity but enjoys the translation-invariant property [45]. Chromosomal patterns of gene expression are not expected to be smooth but to have well defined boundaries. Therefore the WT transform adopts the simplest wavelet, the classic Haar wavelet [46], that allows a constant piece-wise approximation of the RNA profile. As a result, the WT can be described as follows. Firstly, gene expression values obtained from microarray measurements were sorted according to their chromosomal location as previously described. Then the coefficients of the first level of the WT were calculated as the difference of expression between two adjacent probes. The wavelet coefficients of the second level were obtained as the difference between the mean of pairs of adjacent probes. The coefficients of the next levels were then calculated as the differences between the means of P adjacent probes where P increases as a power of 2 (P = 1, 2, 4, 8, ...). The WT is an orthogonal operator and therefore the noise level is identical on the original raw data and at all WT levels. In contrast, clusters of genes with similar level of expression produce a WT coefficient that increases with the resolution level. In other words, genes whose individual expression is undetectable because they are below the noise level are detected through the WT when clustered together because their combined energy condenses into a greater wavelet coefficient.

Note that standard WT is usually applied to equally spaced data, which is not the case here because genes are not equally spaced on chromosomes. Sardy et al. [47] have showed that a wavelet estimator based on the Haar wavelet transform provides an estimate that is at least as good as that recovered by any other Haar wavelet implementation adapted to the unequally spaced case.

### Unsupervised Analysis

Unsupervised analysis was performed using the Singular Value Decomposition algorithm [48] (SVD). SVD was applied to the set of Haar wavelet coefficients [49]. This produced a number of patterns of chromosomal expression equal to the number of the arrays. The contribution of each case to each pattern was calculated as a single number, the "case loading." Case loadings quantify the amount of the pattern expressed by each array. Case loadings were then used for further statistical analysis. Following sections describe the method in detail.:

#### Notation and Haar Wavelet Transform

Let C(h,k) be the matrix for the k = 1, 2, .., M arrays forming the experiment containing on the positions h = 1, 2, ..., 2^n ^the ordered probes. Note that, for algorithmic reasons, the number of probes must be a power of 2 and, if this is not the case, the matrix must be zero-padded. Application of the Haar WT to each column generates n levels of 2^n ^wavelet coefficients that are serially stored in the matrix C_W_(i,k) where i = 1, 2, ..., n2^n^.

#### Individual vs. Global Chromosomal Analysis

Matrix C_W _so far contains the Haar wavelet decomposition for M arrays and one chromosome only. This allows the analysis of each chromosome independently. This option was used in this work for the comparison between individual chromosomal expression and structural changes detected by FISH. However, the core of the work was the analysis of the entire genome simultaneously to detect the combination of chromosomal patterns characteristic of this data-set and, possibly, of gliomas at large. The analysis can be extended to the whole genome by serially adding to the rows of C_W _the Haar wavelet coefficients of all the other chromosomes. The following section is valid for both types of analysis and notation will simply refer to C_W _irrespective of whether it contains the WT of a single chromosome or of all chromosomes.

#### SVD in Wavelet Space

Calculation of SVD for C_W _cannot be solved directly because of the large size of the covariance matrix [C_W_*CWT
 MathType@MTEF@5@5@+=feaafiart1ev1aaatCvAUfKttLearuWrP9MDH5MBPbIqV92AaeXatLxBI9gBaebbnrfifHhDYfgasaacH8akY=wiFfYdH8Gipec8Eeeu0xXdbba9frFj0=OqFfea0dXdd9vqai=hGuQ8kuc9pgc9s8qqaq=dirpe0xb9q8qiLsFr0=vr0=vr0dc8meaabaqaciaacaGaaeqabaqabeGadaaakeaacqqGdbWqdaqhaaWcbaGaee4vaCfabaGaeeivaqfaaaaa@304A@] that has size n2^n ^× n2^n^. This problem can be circumvented by using instead the covariance Cov(C_W_) = CWT
 MathType@MTEF@5@5@+=feaafiart1ev1aaatCvAUfKttLearuWrP9MDH5MBPbIqV92AaeXatLxBI9gBaebbnrfifHhDYfgasaacH8akY=wiFfYdH8Gipec8Eeeu0xXdbba9frFj0=OqFfea0dXdd9vqai=hGuQ8kuc9pgc9s8qqaq=dirpe0xb9q8qiLsFr0=vr0=vr0dc8meaabaqaciaacaGaaeqabaqabeGadaaakeaacqqGdbWqdaqhaaWcbaGaee4vaCfabaGaeeivaqfaaaaa@304A@ * C_W _as follows [48].

Matrix C_W _is firstly normalized by removing the row means and then the SVD is applied to produce the decomposition:

Cov(C_W_) = CWT
 MathType@MTEF@5@5@+=feaafiart1ev1aaatCvAUfKttLearuWrP9MDH5MBPbIqV92AaeXatLxBI9gBaebbnrfifHhDYfgasaacH8akY=wiFfYdH8Gipec8Eeeu0xXdbba9frFj0=OqFfea0dXdd9vqai=hGuQ8kuc9pgc9s8qqaq=dirpe0xb9q8qiLsFr0=vr0=vr0dc8meaabaqaciaacaGaaeqabaqabeGadaaakeaacqqGdbWqdaqhaaWcbaGaee4vaCfabaGaeeivaqfaaaaa@304A@ * C_W _= V_W_S_W_VWT
 MathType@MTEF@5@5@+=feaafiart1ev1aaatCvAUfKttLearuWrP9MDH5MBPbIqV92AaeXatLxBI9gBaebbnrfifHhDYfgasaacH8akY=wiFfYdH8Gipec8Eeeu0xXdbba9frFj0=OqFfea0dXdd9vqai=hGuQ8kuc9pgc9s8qqaq=dirpe0xb9q8qiLsFr0=vr0=vr0dc8meaabaqaciaacaGaaeqabaqabeGadaaakeaacqqGwbGvdaqhaaWcbaGaee4vaCfabaGaeeivaqfaaaaa@3070@     (1)

V_W _is an MxM matrix. Each column [V_W_]_i_, or "singular vector", contains one of the M directions of maximal change for the arrays. These directions are orthogonal to each other and each is made of M coefficients representing the contribution of each array to that particular direction. We label these coefficients as "case loadings."

Case loadings can be used in any type of statistical analysis. For example, they can be entered in a bivariate correlation to test the association of the corresponding chromosomal pattern with clinical parameters by associating the load of each array with the correspondent external measure (say, survival of that particular patient).

S_W _is an MxM diagonal matrix with diagonal elements S_ii _= S_i_. The M diagonal elements S_i _are the singular values of C_W _and, without loss of generality, it can be assumed that they are ordered in decreasing order so that S_1 _≥ S_2 _≥ ... ≥ S_M_. The fraction of total variability in the expression data-set explained by any individual column [V_W_]_i _can be calculated as

*f*[V_W_]_i _= S_i_/Σ (S_i_)     (2)

This means that the first singular vector explains the greatest amount of data variability; the second singular vector contains the direction of change with the second greatest variance and so forth. It is expected that the first singular vectors contain variability due to "real" (biological) signal, while noise contributions will be contained in the last ones [48].

The Haar wavelet patterns corresponding to the singular vectors can be recovered by projecting the matrix C_W _on the rotated axis V_W_.

U_W _= C_W _* V     (3)

Similarly as before, the first columns [U_W_]_i_, corresponding to the first singular vectors, should mostly contain true signal. However a further reduction in the noise can be achieved by removing from matrix U_W _all coefficients smaller than a suitable threshold.

In CHROMOWAVE the filtering procedure incorporates both the noise reduction and the introduction in the model of inter-probe genomic distances according to the procedure described in the next section.

#### Inter-Probes Distance and Noise Penalization

Previous wavelet models of chromosomal gene distribution [20,21] were based on the simplifying assumption that the relation between adjacent probes, if present, does not depend on the absolute physical distance (as measured in base-pairs) but only on contiguity. This assumption may introduce inaccuracies, particularly for local processes involving a very small number of genes because wavelet coefficients (level 2 and above) pool together the expression of probes or groups of probes that may have quite varying distances among them.

In CHROMOWAVE, the likelihood of a Haar wavelet is made proportional to the distance between probes or probe-groups that the wavelet coefficient represents. Inter-probe likelihood was modeled in CROMOWAVE by adding a penalty function to the de-noising procedure.

In wavelet analysis, de-noising is achieved by suppression of all wavelet coefficients that are below an appropriate threshold dependent on the noise levels in the data. This operation requires an estimate of the noise variance of the data. The variances of gene expression measured with microarrays are usually heterogeneous, e.g, vary from gene to gene. However CHROMOWAVE aims to detect gene clusters only; therefore individual gene expressions which correspond to the first level of the WT are of no interest and are suppressed. The other levels of the WT are all generated by pooling together 2,4 8, ..., gene expression values and their variances are, therefore, more homogeneous. Besides, since the WT is an orthogonal operator, all Haar wavelet levels have approximately the same variance that can then be calculated from the robust estimator:

σ^
 MathType@MTEF@5@5@+=feaafiart1ev1aaatCvAUfKttLearuWrP9MDH5MBPbIqV92AaeXatLxBI9gBaebbnrfifHhDYfgasaacH8akY=wiFfYdH8Gipec8Eeeu0xXdbba9frFj0=OqFfea0dXdd9vqai=hGuQ8kuc9pgc9s8qqaq=dirpe0xb9q8qiLsFr0=vr0=vr0dc8meaabaqaciaacaGaaeqabaqabeGadaaakeaacuaHdpWCgaqcaaaa@2E7F@ = MAD(U_W_)/0.6745     (4)

MAD denotes median absolute deviation from 0 and the factor 0.6745 is chosen for calibration with the normal distribution [50].

Spatial modeling and de-noising is therefore achieved by suppressing all coefficients in the matrix U_W _that are under the threshold:

τU=P(w)σ^2log⁡(2n).     (5)
 MathType@MTEF@5@5@+=feaafiart1ev1aaatCvAUfKttLearuWrP9MDH5MBPbIqV92AaeXatLxBI9gBaebbnrfifHhDYfgasaacH8akY=wiFfYdH8Gipec8Eeeu0xXdbba9frFj0=OqFfea0dXdd9vqai=hGuQ8kuc9pgc9s8qqaq=dirpe0xb9q8qiLsFr0=vr0=vr0dc8meaabaqaciaacaGaaeqabaqabeGadaaakeaacqaHepaDdaahaaWcbeqaaiabbwfavbaakiabg2da9iabbcfaqjabcIcaOiabbEha3jabcMcaPiqbeo8aZzaajaWaaOaaaeaacqaIYaGmcyGGSbaBcqGGVbWBcqGGNbWzcqGGOaakcqaIYaGmdaahaaWcbeqaaiabb6gaUbaakiabcMcaPaWcbeaakiabc6caUiaaxMaacaWLjaWaaeWaaeaacqaI1aqnaiaawIcacaGLPaaaaaa@451C@

P(w) is a penalty of the form:

P(w) = 1-G(ln(d),μ,ν)     (6)

In (6), d is the genomic distance between genes or gene groups represented by the Haar wavelet coefficient w and G is the Gaussian cumulative distribution with mean μ and standard deviation ν. This form of the penalty is justified by the fact that inter-wavelet genomic distribution, that we pre-calculated using the available information on the location of the probes, was Gaussian-like for all wavelet levels. Parameters μ and ν were directly obtained by the genomic-alignment information for the HG-U133_Plus_2 chip.

#### Confoundings Due to Aneuploidy and Errors in Normalisation

When applied to the whole chromosome set, the combination of SVD analysis and wavelets has the additional practical utility of identifying errors in the normalisation of data (if linear). With CHROMOWAVE, the application of an inefficient normalization procedure results in a genome-wide constant chromosomal pattern of expression that the SVD identifies and removes from the data. Besides, note that global RNA changes due to aneuploidy also result in the same genome-wide diffuse pattern that can be seemingly removed from the overall data-variability.

#### Chromosomal Pattern Reconstruction

Threshold (5) suppresses all those coefficients that are unlikely to be signal because of their relative height compared to noise and/or because they contain probes/probes clusters that are far apart. All those coefficients surviving the threshold (5) instead are likely contributions to the true signal and are passed through the inverse WT to produce the M filtered patterns of chromosomal variations C_F_(h,k) where h = 1, 2, ..., 2^n ^and k = 1, 2, ..., M.

### Single Profile Generation (Turkheimer et al., 2004 revisited)

CHROMOWAVE allows also the extraction of the differential profile of expression between a single case and a control group (supervised analysis). This application was described previously [20] and is just summarized here. For each chromosome, the differential profile is defined as:

*d*C(i) = C(i) - C¯
 MathType@MTEF@5@5@+=feaafiart1ev1aaatCvAUfKttLearuWrP9MDH5MBPbIqV92AaeXatLxBI9gBaebbnrfifHhDYfgasaacH8akY=wiFfYdH8Gipec8Eeeu0xXdbba9frFj0=OqFfea0dXdd9vqai=hGuQ8kuc9pgc9s8qqaq=dirpe0xb9q8qiLsFr0=vr0=vr0dc8meaabaqaciaacaGaaeqabaqabeGadaaakeaacuqGdbWqgaqeaaaa@2DD1@(i)     (7)

C(i) contains on the positions i = 1, 2, ..., 2^n ^the expressions of the ordered probes for the single case of interest. As before, the number of probes must be a power of 2 and, if this is not the case, the matrix must be zero-padded.

C¯
 MathType@MTEF@5@5@+=feaafiart1ev1aaatCvAUfKttLearuWrP9MDH5MBPbIqV92AaeXatLxBI9gBaebbnrfifHhDYfgasaacH8akY=wiFfYdH8Gipec8Eeeu0xXdbba9frFj0=OqFfea0dXdd9vqai=hGuQ8kuc9pgc9s8qqaq=dirpe0xb9q8qiLsFr0=vr0=vr0dc8meaabaqaciaacaGaaeqabaqabeGadaaakeaacuqGdbWqgaqeaaaa@2DD1@(i) is the average expression of the probes for a control database. Application of the WT to *d*C(i) generates n levels of 2^n ^wavelet coefficients that are serially stored in the matrix *d*C_W_(i,j). Differently from before i = 1, 2, ..., 2^n ^indexes now 2^n ^locations and j = 1, 2, ..., n indexes the n wavelet resolutions. The differential profile is de-noised by suppression of the coefficients below the threshold defined in equation (5) where the penalty P(w) is the same as in equation (6) and the variance σ^
 MathType@MTEF@5@5@+=feaafiart1ev1aaatCvAUfKttLearuWrP9MDH5MBPbIqV92AaeXatLxBI9gBaebbnrfifHhDYfgasaacH8akY=wiFfYdH8Gipec8Eeeu0xXdbba9frFj0=OqFfea0dXdd9vqai=hGuQ8kuc9pgc9s8qqaq=dirpe0xb9q8qiLsFr0=vr0=vr0dc8meaabaqaciaacaGaaeqabaqabeGadaaakeaacuaHdpWCgaqcaaaa@2E7F@ is calculate as

σ^
 MathType@MTEF@5@5@+=feaafiart1ev1aaatCvAUfKttLearuWrP9MDH5MBPbIqV92AaeXatLxBI9gBaebbnrfifHhDYfgasaacH8akY=wiFfYdH8Gipec8Eeeu0xXdbba9frFj0=OqFfea0dXdd9vqai=hGuQ8kuc9pgc9s8qqaq=dirpe0xb9q8qiLsFr0=vr0=vr0dc8meaabaqaciaacaGaaeqabaqabeGadaaakeaacuaHdpWCgaqcaaaa@2E7F@ = MAD(*d*C_W_(i,1))/0.6745     (8)

*d*C_W_(i,1) is the finest resolution level of the wavelet transform.

Upon application of the inverse wavelet transform to the filtered matrix *d*C_W_(i,j) one obtains a de-noised approximation of the individual pattern *d*C(i).

We used CHROMOWAVE to extract patterns of chromosomal expression for individual tumour cases by contrasting its microarray measurement with the average expression of a normal database of 11 normal brain samples. RNA extracted from these cases was hybridized to Affymetrix U133_Plus_2 arrays and data processed as described previously.

### Fluorescence in situ hybridization (FISH)

The 27 tumours were all also studied with FISH. Dual-colour assays were performed on 8-μm-thick cryostatic sections from the same tissue blocks as those used for microarray experiments. We tested the six chromosomes that revealed the major changes with CHROMOWAVE. Loss or gain on chromosomes 1 and 19 were detected with LSI^®^1p36/LSI 1q25 and LSI 19q13/LSI 19p13 dual-color probe sets (Vysis, Inc., Downers Grove, IL, USA). Chromosome 9q was studied with the ABL (9q34) probe of the LSI ABL/BCR ES probe system (Vysis). For chromosomes 4, 13, 15 and 18, we used Vysis probes CEP4, LSI13 (440 kb including the RB gene in 13q14), CEP15 and CEP18. Samples were processed according to the manufacturer's protocol. Results were evaluated with an Olympus BX51 fluorescence microscope (Olympus, Melville, NY, USA) equipped with the appropriate fluorescence filters. At least 100 cells were examined for all signals and the mean signal numbers were recorded. Frozen sections of normal brain were analysed to establish a reference FISH copy number. Upper and Lower normality thresholds were calculated as mean +/- 2 standard deviations (SD). Tumour samples with mean signal below the lower threshold were reported as showing monosomy. Correlation between FISH mean copy numbers and CHROMOWAVE loadings were calculated using Pearson product moment correlation coefficient.

## Availability and Requirements

The microarray data used in this work are deposited in the GEO database (GEO Submission GSE2817). Software available on request, free for academic users.

Project name: CHROMOWAVE

Project home page: 

Operating system(s): Platform independent;

Programming language: Matlab 7 (R14)

Licence: GNU

Non-academics: licence needed.

## Authors' contributions

FET developed the methodology, the code, performed part of the data-analysis and drafted the manuscript. FR participated in the design of the study, selected the cases and contributed to the manuscript. BH and VB carried out the RNA extraction, microarray processing and helped with data analysis. AE and CH performed the FISH studies and aided the data analysis and interpretation. MN and DM were responsible for the clinical data. JB participated in the study design and in the preparation of the manuscript. MD developed the study design and drafted the manuscript. All authors read and approved the final manuscript.

## Supplementary Material

Additional File 1 Data for Figure 1. This table details the pattern illustrated in Figure 1. The file contains data sequentially for the chromosomes and consists of 5 columns The first one contains the Affymetrix probe ID, the second one is the expression (log2 scale), the third one contains the chromosomal locus, the 4th one is the exact genome distance for that chromosome (Zero is the p telomere in Affymetrix convention). Column 5 contains the ontology. Additional information is provided in form of colours as follows: - In yellow clusters with log2 expression > abs(0.8) with Citation in PubMed under keyword "GLIOMA" - In orange genes with citation in OMIM under keyword "TUMOUR" (chr4, chr13, chr15, chr18, chrY only) - In red genes with citation in OMIM under keyword "Glioma" (all chromosomes) but not necessarily with high or low expression in this data set. Click here for file
